# Diagnostic Performance of Angiography-Based Fractional Flow Reserve for Functional Evaluation of Coronary Artery Stenosis

**DOI:** 10.3389/fcvm.2021.714077

**Published:** 2021-10-12

**Authors:** Changling Li, Xiaochang Leng, Jingsong He, Yongqing Xia, Wenbing Jiang, Yibin Pan, Liang Dong, Yong Sun, Xinyang Hu, Jian'an Wang, Jianping Xiang, Jun Jiang

**Affiliations:** ^1^Department of Cardiology, The Second Affiliated Hospital, Zhejiang University School of Medicine, Hangzhou, China; ^2^ArteryFlow Technology Co., Ltd., Hangzhou, China; ^3^Department of Cardiology, The Third Clinical Institute Affiliated to Wenzhou Medical University, Wenzhou, China; ^4^Department of Cardiology, Affiliated Jinhua Hospital, Zhejiang University School of Medicine, Jinhua, China

**Keywords:** fractional flow reserve, invasive coronary angiography, coronary artery, ischemia, stenosis

## Abstract

**Background:** A new method for calculating fraction flow reserve (FFR) without pressure-wire (angiography-derived FFR) based on invasive coronary angiography (ICA) images can be used to evaluate the functional problems of coronary stenosis.

**Objective:** The aim of this study was to assess the diagnostic performance of a novel method of calculating the FFR compared to wire-based FFR using retrospectively collected data from patients with stable angina.

**Methods:** Three hundred patients with stable angina pectoris who underwent ICA and FFR measurement were included in this study. Two ICA images with projections >25° apart at the end-diastolic frame were selected for 3D reconstruction. Then, the contrast frame count was performed in an angiographic run to calculate the flow velocity. Based on the segmented vessel, calculated velocity, and aortic pressure, AccuFFRangio distribution was calculated through the pressure drop equation.

**Results:** Using FFR ≤ 0.8 as a reference, we evaluated AccuFFRangio performance for 300 patients with its accuracy, sensitivity, specificity, positive predictive value (PPV), and negative predictive value (NPV). Comparison of AccuFFRangio with wire-measured FFR resulted in an area under the curve (AUC) of 0.954 (per-vessel, *p* < 0.0001). Accuracy for AccuFFRangio was 93.7% for Pa set from measurement and 87% for Pa = 100 mmHg in this clinical study. Overall sensitivity, specificity, PPV, and NPV for per-vessel were 90, 95, 86.7, 96.3, and 57.5, 97.7, 90.2, 86.3%, respectively. Overall accuracy, sensitivity, specificity, PPV, and NPV for 2-dimensional (2D) quantitative coronary angiography (QCA) were 63.3, 42.5, 70.9, 34.7, and 77.2%, respectively. The average processing time of AccuFFRangio was 4.30 ± 1.87 min.

**Conclusions:** AccuFFRangio computed from coronary ICA images can be an accurate and time-efficient computational tool for detecting lesion-specific ischemia of coronary artery stenosis.

## Introduction

Compared with the anatomical stenosis of the coronary artery, functional assessment can more accurately evaluate and predict the progression of coronary heart disease (1). In the catheterization laboratory, invasive coronary angiography (ICA) images can only qualitatively assess the degree of stenosis but cannot evaluate the physiological function of coronary arteries. Therefore, it may overestimate or underestimate the severity of the disease, leading to the untreated or over-treatment of lesions (2). Fractional flow reserve (FFR) has become a recognized index for the functional evaluation of coronary stenosis, which is defined as a ratio of the pressure of the distal end of the stenosis and the cardiac aorta at hyperemia (1). The current method of measuring FFR requires a pressure wire inserted into the distal end of the stenosis, which will bring additional procedure-related risks causing adverse effects to the blood vessel and increase the treatment time and cost (3, 4). A new method of non-pressure wire FFR (angio-based FFR) calculation method based on ICA images can reflect functional problems of coronary stenosis (5–7). By using two angiograms greater than 25° through independent 3D vessel reconstruction and numerical calculation of pressure drop, angio-based FFR enables interventional cardiologists and researchers to obtain accurate anatomical quantifications of one or more lesions in the analyzed coronary segment, to determine the functional significance of the individual and consecutive multiple lesions. These methods can be helpful for optimal percutaneous coronary intervention (PCI) treatment of the lesion of coronary disease. Several studies have shown that angio-based FFR is highly correlated with invasive FFR compared to coronary computed tomography angiography (CTA) and ICA assessment (4, 8–10). It is also more advantageous in formulating treatment strategies for coronary artery disease under circumstances that screening people with suspected chest pain for the presence of myocardial ischemia.

In this study, coronary angiography was used to calculate the average volume flow using TIMI (thrombolysis in myocardial infarction) frame count combined with three-dimensional quantitative coronary angiography (QCA). Subsequently, applying computational fluid dynamics theory, a new angiography-based FFR calculation method AccuFFRangio was proposed. The FFR measured by the pressure-wire was used as a reference standard to evaluate the diagnostic performance of AccuFFRangio.

## Materials and Methods

### Study Design

The present study is a retrospective, single-center, observational study performed at The Second Affiliated Hospital, Zhejiang University School of Medicine. This study aims to evaluate the diagnostic accuracy, sensitivity, and specificity of AccuFFRangio in identifying functionally significant stenosis by using pressure wire-based FFR as the reference. AccuFFRangio and 2D-QCA were analyzed and compared in the core laboratory of the Department of Cardiology at The Second Affiliated Hospital, Zhejiang University School of Medicine. After receiving ethics approval from the institutional review board, this study was conducted with a written informed consent form waived.

### Patient Population

Since this was a retrospective study, consecutive patients with stable angina pectoris who underwent ICA and FFR measurement were eligible for enrollment. Angiographic inclusion criteria were (1) percentage diameter stenosis of the coronary artery between 30% and 90% in a vessel ≥2 mm by visual estimation; (2) angiographic projections ≥25° apart were recorded. Exclusion criteria include (1) overlapping interrogated vessels with too much shortening without preferred references in proximal or distal vessels; (2) insufficient injected contrast for QCA analysis; (3) location of the target lesion at the ostium of the left or right coronary artery; (4) wire-position not documented. Exclusion criteria on patient level contain (1) acute myocardial infarction within 72 h; (2) severe asthma or severe chronic obstructive pulmonary disease; (3) allergy to contrast media or adenosine; or (4) atrial fibrillation.

### Invasive Coronary Angiography and 2D-QCA Analysis

ICA was performed using the X-ray system (Allura Xper FD20/10; PHILIPS Medical Systems, the Netherlands). These angiographic images were recorded at 15 frames/s. The contrast medium was injected manually with a forceful and stable injection or by the pump at a rate of ~4 ml/s. 2D-QCA was conducted by using angiogram vendor-integrated QCA software (Allura Xper FD20/10; PHILIPS Medical Systems, the Netherlands).

### Wire-Based FFR Measurement

**Figure 1:**
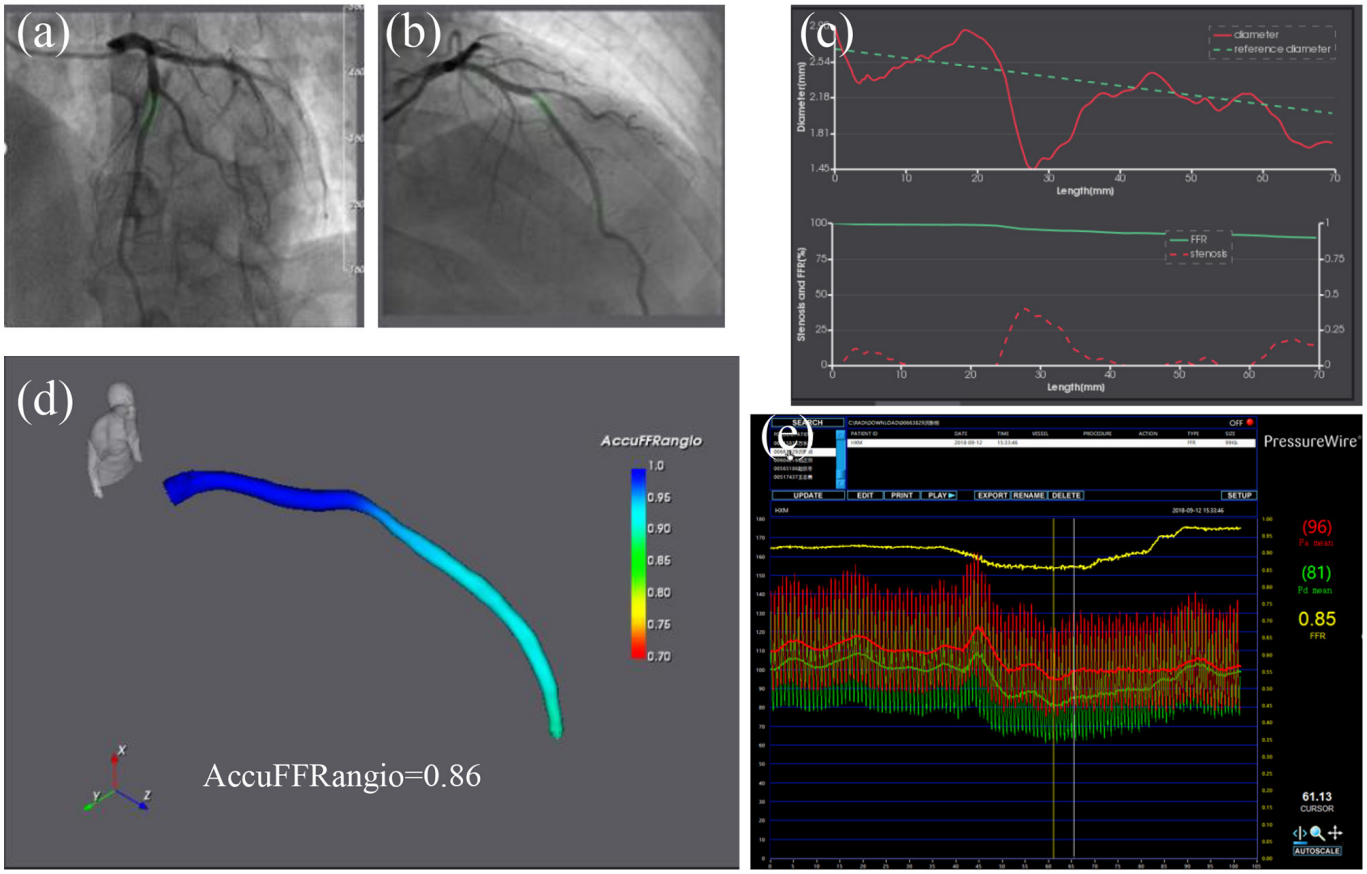
AccuFFRangio analysis of intermediate stenosis of a left anterior descending artery (LAD). **(a,b)** Coronary angiography images from two different angles of view. **(c)** The above chart shows the change of lumen diameter (red) along LAD with the computed reference diameter (green); the chart below shows the diameter stenosis (red) and AccuFFRangio pullback (green). **(d)** Computed AccuFFRangio distribution; the AccuFFRangio value was 0.86. **(e)** The FFR measured by pressure wire was 0.85.

FFR was measured in all patients using coronary pressure wire (St. Jude Medical, St. Paul, MN, USA). After calibration and equalization, the pressure wire was advanced distally to the stenosis. Maximum hyperemia was induced with i.v. adenosine triphosphate at a concentration of 180 μg/kg/min. Both the distal coronary pressure at the pressure sensor and the proximal pressure at the coronary artery ostium were recorded simultaneously. The FFR measurement was performed by physicians in The Second Affiliated Hospital, Zhejiang University School of Medicine (Y.P., L.D., W.J., Y.S.). Pressure sensor was pulled back to the catheter tip to check or correct the pressure drift (Figure 1e).

### AccuFFRangio Computation

AccuFFRagnio was computed with the AccuFFRangio V1.0 software (ArteryFlow Technology, Hangzhou, China) by participating physicians and technicians (F.M., Y.Z., M.H.) blinded to FFR. Two angiographic images with projections >25° apart at the end-diastolic frame were selected for three-dimensional (3D) reconstruction (6, 7, 11). To simplify the geometry calibration procedure and achieve a reliable correspondence in centerline points for 3D reconstruction, we have implemented three pairs of reference points to eliminate the isocenter offset and rotational angle parameter errors. Since the pressure drop has a positive relationship with the coronary vessel flow rate, the frame count method is a relatively feasible solution (12). This method hypothesizes that the blood flow velocity is proportional to the vessel cross-section diameter dimension. Typically, the pressure drop from proximal to distal stenosis is caused by two factors. The first is the viscous pressure drop associated with friction. The second is the expansion pressure drop due to the rapid change in radius, which is usually characterized by narrowing. Pressure drop *P*_*R*_ is related to viscosity loss coefficient *C*_*Vis*_, expansion loss coefficient *C*_*Expan*_, and flow rate *Q*: *P*_*R*_ = (*C*_*Vis*_ + *C*_*Expan*_ ∙ *Q*) ∙ *Q*. More detail of the derived equations can be seen in our previous study (11).

Contrast flow rate velocity for FFR computation was derived from the TIMI frame counting method for the segmented vessel. With the calculated velocity and input aortic pressure from the measurement of the pressure at the coronary ostium, AccuFFRangio distribution can be calculated (Figure 1). AccuFFRangio value was taken at the same position of wire-based FFR using angiography images as a reference. To compare the diagnostic accuracy of AccuFFRangio by using a fixed value of aortic pressure and to study the influence of fixed value on the performance of our approach in case some patient-specific pressures cannot be obtained, Pa = 100 mmHg was set for each calculation of angio-based FFR (13, 14).

### Statistical Analysis

Continuous variables with normally distributed were expressed as mean ± standard deviation (SD) and non-normal distributed variables as the median. Categorical variables were expressed as percentages and data were analyzed on a per vessel basis. Pearson correlation was used to quantify the correlation between FFR and AccuFFRangio. Agreement between FFR and AccuFFRangio was assessed on the Bland-Altman plot. Using FFR ≤ 0.8 as the reference standard, the performance of AccuFFRangio for predicting functionally significant stenosis was evaluated by diagnostic accuracy, sensitivity, specificity, positive predictive value (PPV), and negative predictive value (NPV). The area under the curve (AUC) of receiver operating characteristic (ROC) analysis was used to assess the diagnostic accuracy of AccuFFRangio. All the statistical analyses were performed by using MedCalc (MedCalc Software Inc., Belgium).

## Results

### Patient Characteristics

**Figure 2:**
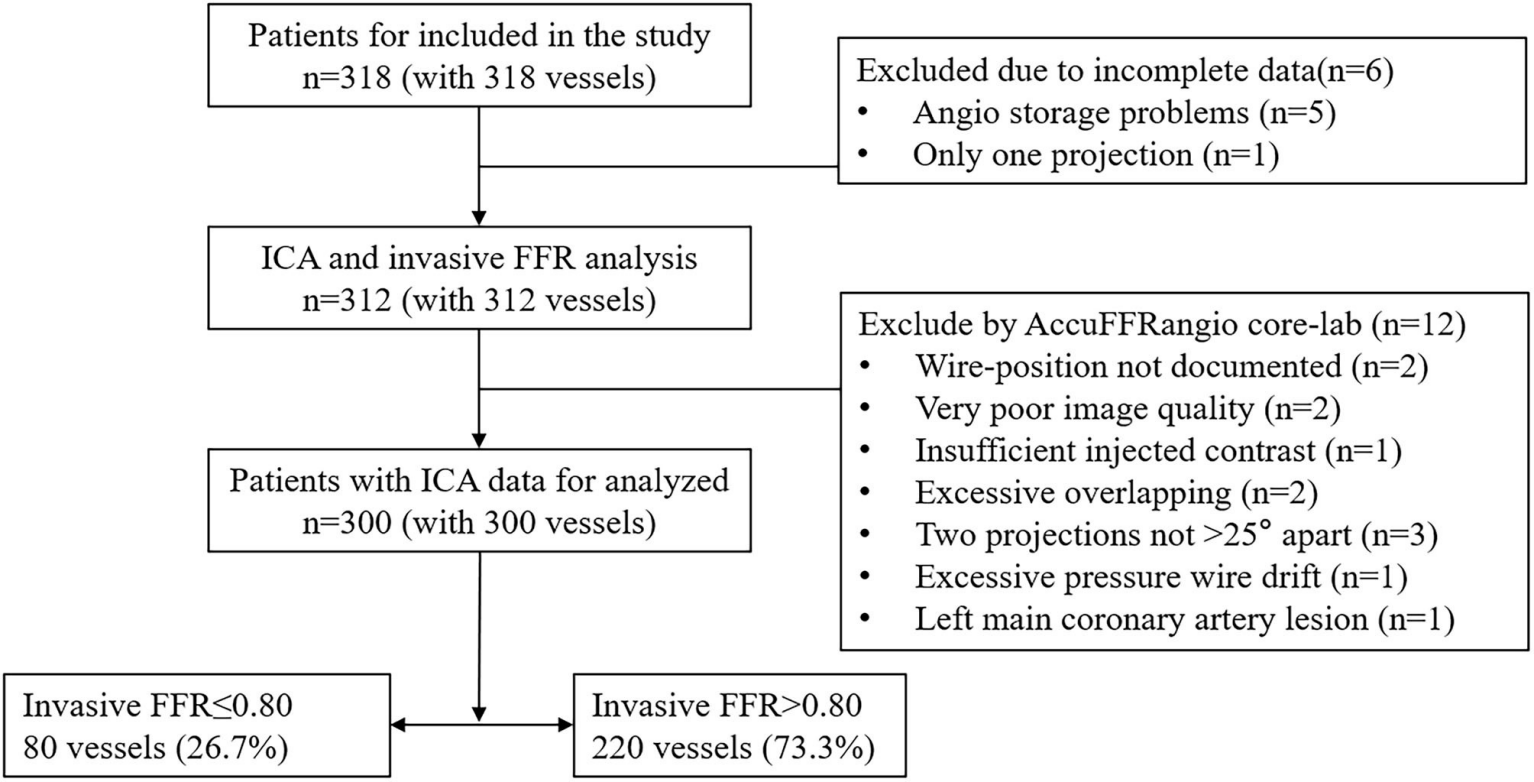
Study enrollment flow chart.

Figure 2 presents the study enrollment flow chart. Three hundred eighteen patients with 318 vessels were included in this clinical study from May 2016 to July 2019. Due to the incomplete data from six patients, 312 patients underwent ICA procedure and wire-FFR waveform analysis. Among them, 12 patients were excluded due to predefined exclusion criteria, including undocumented wire-position, poor image quality, excessive vessel overlap, insufficient contrast, projections <25 degrees, excessive pressure wire drift, and left main coronary artery lesions. In the end, 300 patients with 300 vessels were included in the final analysis.

**Figure 3:**
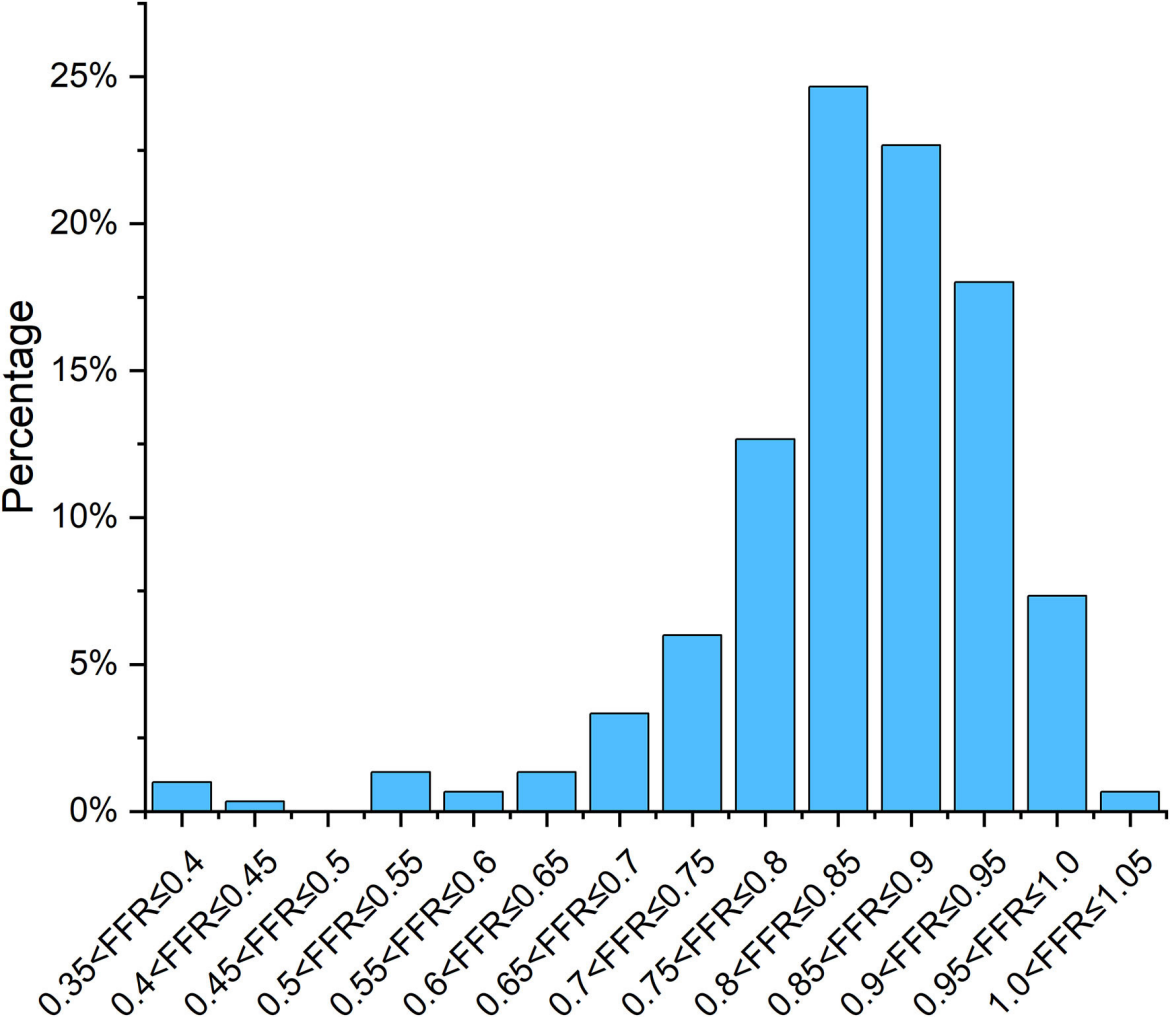
Percent distribution of invasive FFR.

Mean FFR was 0.84 ± 0.10, as shown in Figure 3, and mean percentage diameter stenosis (%DS) form 2D-QCA was 44 ± 12%. FFR ≤ 0.80 was found in 80 (26.7%) vessels and the mean contrast flow rate velocity was 0.17 ± 0.05 m/s. Baseline patient and procedural characteristics are listed in Tables 1, 2.

**Table 1:** Baseline patient characteristics (*n* = 300).

Age, y	64.1 ± 9.6
Male	67% (201)
Weight (kg)	68.5 ± 34.5
Height (cm)	165 ± 7.3
BMI, kg/m^2^	25.2 ± 13.9
Systolic blood pressure (mm Hg)	130 ± 20
Diastolic blood pressure (mm Hg)	78 ± 15
Diabetes	21% (64)
Hypertension	45% (135)
Hyperlipidemia	13% (40)

**Table 2:** Vessel characteristics (*n* = 300).

**Vessels**	
LAD	61.7% (185)
LCX	7.3% (22)
RCA	29.7% (89)
**Anatomy**	
Diameter stenosis, %	44 ± 12%
<50%	67.3% (202)
≥50%	32.7% (98)
**Physiology**	
FFR (per vessel)	0.84 ± 0.10
Vessels with FFR ≤ 0.8	26.7% (80)
Vessels with FFR > 0.8	73.3% (220)
Diffuse or serial lesions	32.3% (97)
Bifurcation lesions	2.7% (8)
Calcified lesions	2% (6)
Myocardial bridge	5.7% (17)

### Correlation and Agreement Between AccuFFRangio and FFR

**Figure 4:**
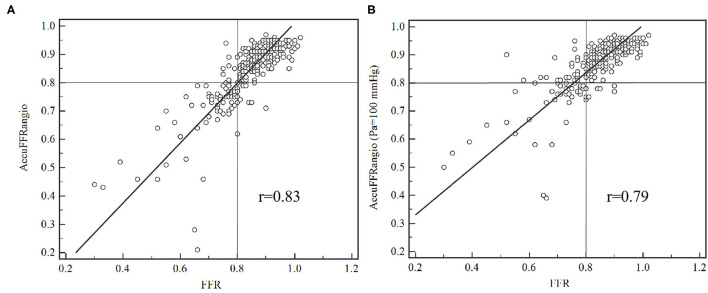
Correlation between AccuFFRangio computation and conventional pressure wired measured FFR. **(A)** Pa from the measurement of the pressure at the coronary ostium; **(B)** Pa set as equal to 100 mmHg.

**Figure 5:**
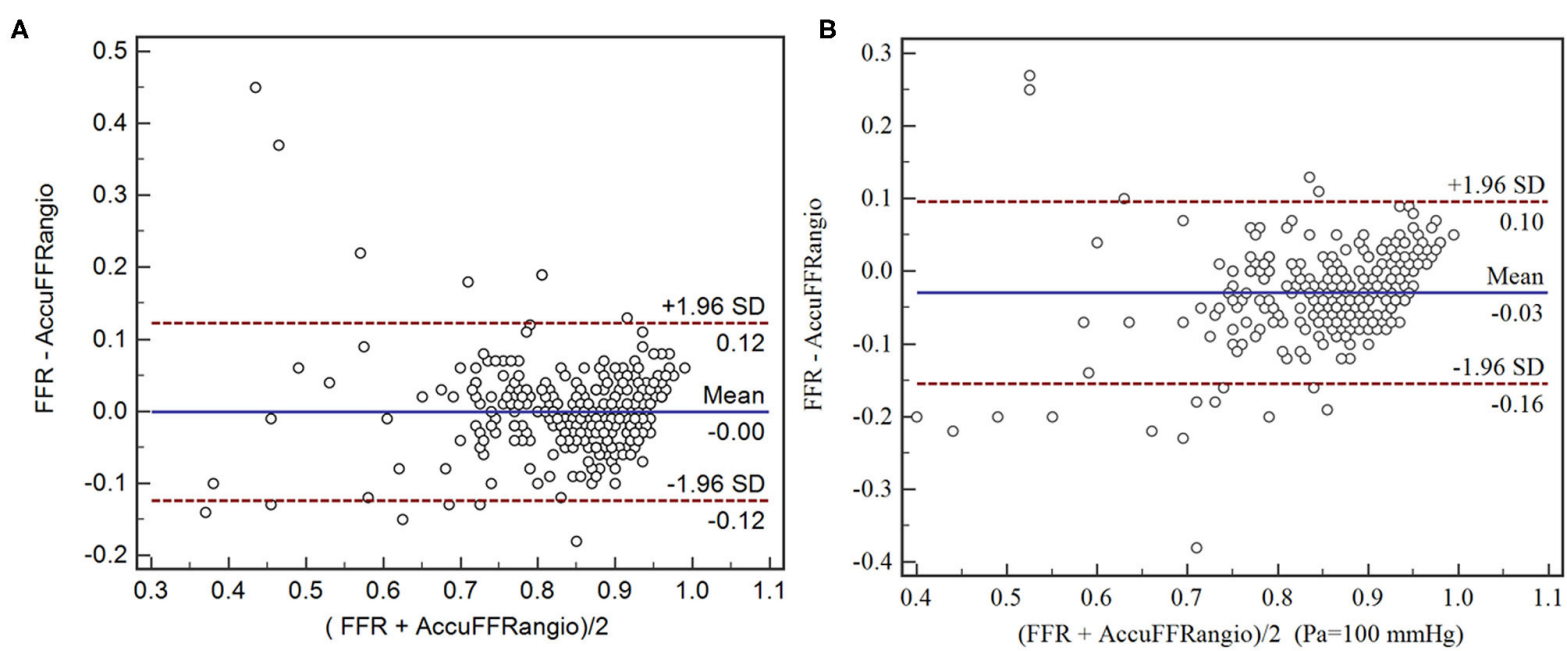
Agreement between AccuFFRangio computation and conventional pressure wired measured FFR. **(A)** Pa from the measurement of the pressure at the coronary ostium; **(B)** Pa set as equal to 100 mmHg.

Good correlations were observed in Figure 4 with a correlation coefficient of *r* = 0.83 (*p* < 0.001). There were good agreements between AccuFFRangio and FFR in the Bland-Altman plot with a mean difference value of −0.001 (limits of agreement: −0.124 to 0.122) when Pa measured at the coronary ostium and −0.030 (limits of agreement: −0.155 to 0.095) when Pa = 100 mmHg was used, as shown in Figure 5. The number of patients with the absolute difference between AccuFFRangio and FFR falling out of the 95% CI was 9 (3%) when Pa was set according to the patients and 18 (6%) when Pa was set equal to a fixed value.

### Diagnostic Performance of AccuFFRangio and 2D-QCA

**Figure 6:**
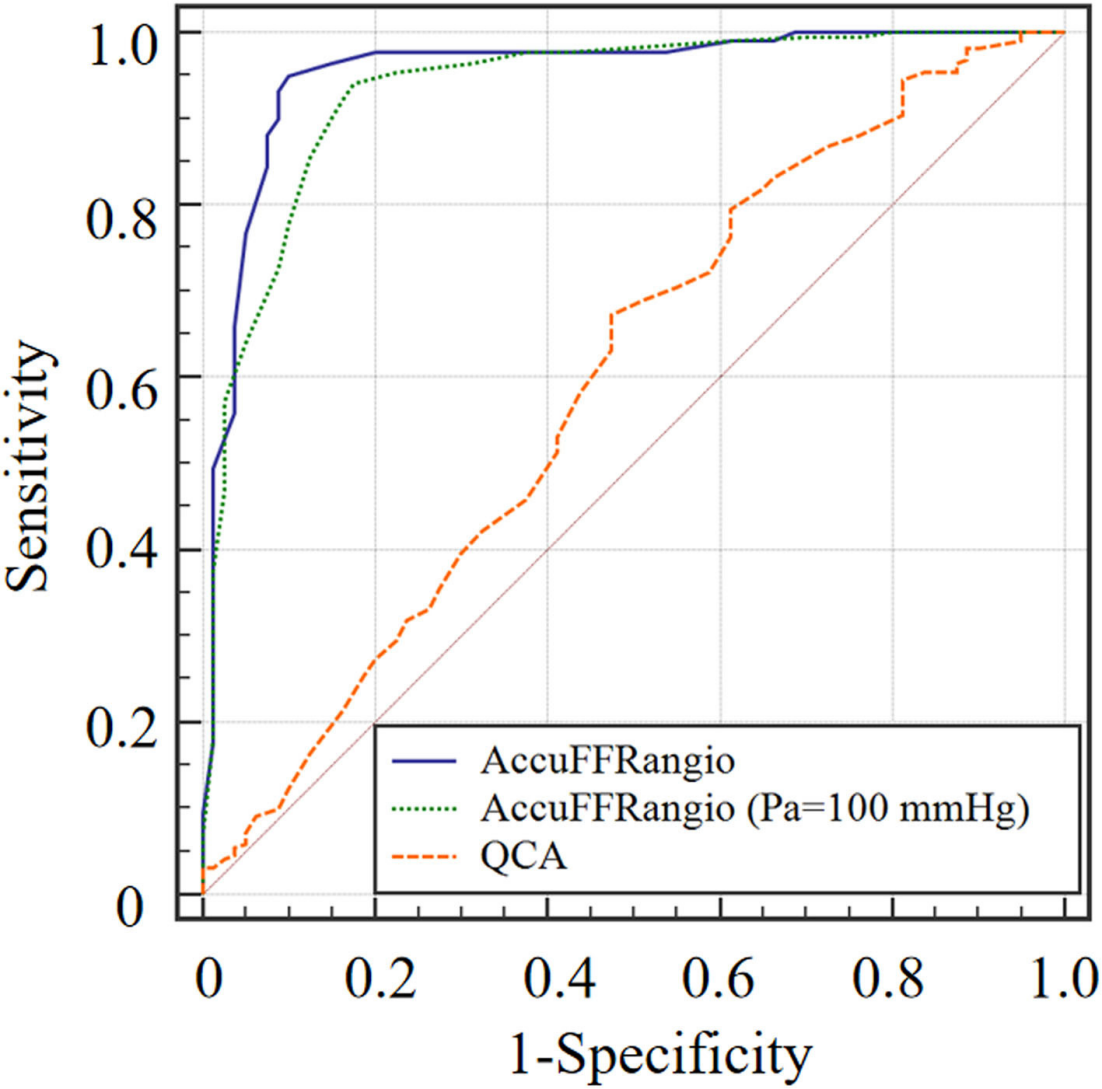
ROC Curve between AccuFFRangio and QCA.

Accuracy for AccuFFRangio was 93.7% in this clinical study. Overall sensitivity, specificity, PPV, and NPV were 90, 95, 86.7, and 96.3%, respectively (Table 3). Meanwhile, these values for AccuFFRangio, when Pa = 100 mmHg was implemented in the calculation, were 87, 57.5, 97.7, 90.2, and 86.3%. Comparison of AccuFFRangio and 2D-QCA with pressure wire measured FFR as reference resulted in an AUC for AccuFFRangio of 0.954 (95%CI: 0.924–0.975) and 0.934 (95%CI: 0.900–0.960, when Pa = 100 mmHg) and 2D-QCA of 0.567 (95% CI: 0.509–0.624), as shown in Figure 6.

**Table 3:** Diagnostic performance of AccuFFRangio for per-vessel.

	**AccuFFRangio ≤ 0.8**	**AccuFFRangio ≤ 0.8 (Pa = 100 mmHg)**	**Diameter stenosis by QCA ≥ 50%**
Accuracy	93.7% (89.9–95.9%)	87% (81.9–90.0%)	63.3% (57.94–69.1%)
Sensitivity	90.0% (84.6–97.2%)	57.5% (45.3–67.8%)	42.5% (33.8–56.5%)
Specificity	95.0% (89.5–96.5%)	97.7% (94.1–99.0%)	70.9% (63.9–76.4%)
PPV	86.7% (76.3–89.9%)	90.2% (77.3–94.5%)	34.7% (28.8–43.2%)
NPV	96.3% (94.1–98.7%)	86.3% (82.6–88.7%)	77.2% (73.9–81.4%)

*Data are shown in percentage with 95% confidence interval in brackets*.

In addition, the mean processing time of AccuFFRangio assessment was 4.30 ± 1.87 min including the 3D anatomic model reconstruction and AccuFFRangio calculation for each patient.

## Discussion

Wire-based FFR has potential risks in measurement procedures and vasodilator complications, and the complexity of the operation is also a challenge. In this situation, this study had demonstrated a reliable and efficient computational method AccuFFRangio for the functional evaluation of lesion-specific ischemia of coronary artery stenosis based on ICA images without injecting vasodilators. Thus, instead of using invasive wire-based FFR for evaluating the severity of suspected coronary heart disease, AccuFFRangio uses a combination of the 3D structure of the coronary vessel and computational fluid dynamics (CFD)-based equations on account of TIMI frame count to analyze the functional performance in a short time of 5 min. The diagnostic accuracy of AccuFFRangio was 93.7% compared to pressure wire-derived FFR, which shows a higher accuracy compared to 2D-QCA with an accuracy of 63.3%.

For assessment of FFR without pressure-wire, many research groups have made significant efforts and developed different angiography-based FFR methods. Morris et al. (15) described that the construction of arteries was based on two projections from similar phases of the cardiac cycle with good vessel opacification and contrast. Meanwhile, the virtual FFR was calculated from CFD simulations with generic downstream boundary conditions applied to the arterial outlet with a Windkessel model (16). With a 3D coronary tree construction based on the geometry of two or more projections with a minimum separation of 30° and application of an automatic resistance-based lumped model of the entire coronary tree, FFR_angio_ (17–19) showed a high concordance between pressure-wire measured FFR. By reconstructing a 3D QCA model of the target vessel using two angiographic projections recorded at least 25° intervals, the QFR was computed through mathematic equations incorporated with contrast flow velocity determined using frame count analysis (6, 20). It represented a high diagnostic accuracy with FFR, reducing the number of patients for pressure-wire measurements. In the current study of AccuFFRangio, the 3D geometry model construction and calculation of FFR were different from the methods described above. We used three physiological points to do the vessel calibration to eliminate the error during the reprojection procedure, as the same processes in our previous studies (11). Moreover, the velocity of the inlet of the blood vessel was set according to the TIMI frame count. The blood pressure at the aorta was chosen equal to the value from measuring the pressure at the coronary ostium.

The accuracy of AccuFFRangio in the present study was 93.7% for per-vessel bias, which is comparable to the clinical trials with patients over 200, such as 83% for WIFI II Study (21), 86.8% for FAVOR II Europe-Japan Study (7), 92.7% for FAVOR II China Study (4), and 93.5% for FAST-FFR Study (18). The sensitivity, specificity, and AUC for the four clinical trials were 86, 77%, and 0.86 for WIFI II Study (21), 86.5, 86.9%, and 0.92 for FAVOR II Europe-Japan Study (7), 94.6, 91.7%, and 0.96 for FAVOR II China Study (4), and 91.2, 92.2%, and 0.944 for FAST-FFR Study (18). Those for AccuFFRangio were 90, 95%, and 0.954. Compared to Pa taken from the measurement at the coronary ostium, as Pa set equal to a fixed value of 100 mmHg, the diagnostic performance decreased to 87% for accuracy and 57.5% for sensitivity, respectively. Angiography-based FFR can improve the low sensitivity of 2D-QCA in evaluating hemodynamically significant of coronary stenosis, from about 42.5–62.5–86.5–94.6% (4, 7, 21–23). Similarly, this method will also increase the specificity from the original 58.1–76.5–86.9–92.2% (4, 7, 21–23). Thus, the implementation of angiography-based FFR can avoid unnecessary revascularization of many interrogated vessels when performed coronary angiography. It is also useful to optimize the strategies of percutaneous coronary intervention (PCI) to reduce the number of the implanted stents and improve the clinical outcome for patients who plan to perform PCI.

The time for calculating angiography-based FFR is also essential for evaluating superiority when there is only limited time during the PCI operation. For vFFR (15), it took ~ 24 h for the CFD simulation of one case, which cannot be implemented in the condition during PCI performance. Another method FFR_angio_ took nearly 10 min for the whole procedure, including reconstruction of the 3D geometry model of the entire coronary tree and calculation of the FFR values based on lumped model (17, 18, 24, 25). But, it only took 4.3 ± 3.4 min to perform an analysis for one lesion (24). By constructing the 3D geometry model for only the target vessel and CFD-based equations on account of TIMI frame count to calculate the FFR values, the entire procedure was completed in a short time of 5 min 59 s on average (6), which can be used during the PCI operation. In addition, the entire process of calculating FFR with a similar algorithm used in this paper took about 5 min, which met the requirement of clinical application in PCI surgery.

It is worth noting the limitations of this clinical study. Firstly, this was a retrospective study at one single center. Secondly, the study may have selection bias because of the relatively small number of positive cases (80 vessels, 26.7%) compared with the negative ones (220 vessels, 73.3%). Third, this study was an observational study. In the future, prospective, multi-center, and follow-up studies will be performed in the post-market clinical researches. Fourth, abnormal pressure curves such as wave form distortion or ventricularization were not found in this study due to the nature of our study population, while this could influence the measurement of FFR; thus, further assessment should be considered.

## Conclusions

This clinical study demonstrates that AccuFFRangio is clinically feasible. The performance is superior to angiographic assessment by 2D-QCA for evaluating coronary artery stenosis when using FFR as a reference. The accuracy, sensitivity, and specificity of AccuFFRangio in identifying hemodynamically significant of coronary stenosis using 300 patient data were 93.7, 90, and 95%, respectively. Those were better than the diagnostic performance of AccuFFRangio calculated based on Pa setting equal to 100 mmHg. AccuFFRangio bears the potential of improving angiography-based identification of functionally significant stenosis during coronary angiography procedure.

### Impact on Daily Practice

AccuFFRangio can quickly and accurately calculate FFR values based on coronary angiography images and can be used for functional assessment of patients with coronary heart disease, avoiding unnecessary PCI treatment.

## Data Availability Statement

The original contributions presented in the study are included in the article/supplementary material, further inquiries can be directed to the corresponding authors.

## Ethics Statement

The studies involving human participants were reviewed and approved by Department of Cardiology, Second Affiliated Hospital of Zhejiang University School of Medicine. Written informed consent for participation was not required for this study in accordance with the national legislation and the institutional requirements.

## Author Contributions

CL and XL: concept and design of the study, analysis of the data, and drafting of the manuscript. JH: case calculation and drafting of the manuscript. CL, LD, and XH: image annotation of ICA and critical review of the manuscript. YX and JH: implementation of the algorithm and drafting of the manuscript. JW and JX: conception and design of the study, analysis of the data, drafting of the manuscript, and final approval of the manuscript submitted. WJ, YP, YS, and JJ: have contributed to the submitted work. All authors contributed to the article and approved the submitted version.

## Funding

This work was supported by the National Natural Science Foundation of China (Nos. 82170332, 81320108003, 31371498, 81100141, and 81570322), Zhejiang Provincial Public Welfare Technology Research Project (No. LGF20H020012), Zhejiang Provincial key research and development plan (No. 2020C03016), the Major projects in Wenzhou of China (No. 2019ZG0107), the Major projects in Jinhua of China (No. 2020A31003), Scientific research project of Zhejiang Education Department (No. Y201330290), and Major medical and health science and technology plan of Zhejiang Province (No. WKJ-ZJ-1913).

## Conflict of Interest

XL is employed by ArteryFlow Technology and receives grants from National Natural Science Foundation of China (No. 11802113). JH and YX was employed by ArteryFlow Technology. JX is the CEO of ArteryFlow Technology and receives grants from National Natural Science Foundation of China (No. 81771242). The remaining authors declare that the research was conducted in the absence of any commercial or financial relationships that could be construed as a potential conflict of interest.

## Publisher's Note

All claims expressed in this article are solely those of the authors and do not necessarily represent those of their affiliated organizations, or those of the publisher, the editors and the reviewers. Any product that may be evaluated in this article, or claim that may be made by its manufacturer, is not guaranteed or endorsed by the publisher.
